# Changes in Reconstructed Soil Physicochemical Properties in an Opencast Mine Dump in the Loess Plateau Area of China

**DOI:** 10.3390/ijerph19020706

**Published:** 2022-01-09

**Authors:** Yuting Li, Wenxiang Zhou, Ming Jing, Shufei Wang, Yuhan Huang, Bingjin Geng, Yingui Cao

**Affiliations:** 1 School of Land Science and Technology, China University of Geosciences, Beijing 100083, China; 2012190028@cugb.edu.cn (Y.L.); zhouwenxiang@email.cugb.edu.cn (W.Z.); 3012200010@cugb.edu.cn (S.W.); 3012190017@cugb.edu.cn (Y.H.); 2012190030@cugb.edu.cn (B.G.); 2 China Natural Resources News, Beijing 100860, China; 3 Key Lab of Land Consolidation and Rehabilitation, Ministry of Natural Resources of the PRC, Beijing 100035, China

**Keywords:** land reclamation, Loess Plateau area, reconstructed soils, physicochemical properties, revegetation

## Abstract

Soil construction and revegetation are essential for ecological restoration in mining areas. The influence of vegetation on the horizontal and vertical distribution patterns of soil properties should be fully understood. However, most studies on reconstructed soils in mining areas only concentrate on the surface soil, without exploring the vertical variations in soil properties. Overall, this study aims to explore the potential mechanisms by which surface vegetation exerts some influence on the spatial distribution of soil physicochemical properties, and to provide some insight into revegetation and soil reclamation in mining areas. Descriptive statistics and one-way analysis of variance (one-way ANOVA) were employed to evaluate the differences in the soil physicochemical properties in horizontal and vertical directions under different land-use types in the south dump of Antaibao opencast mine in Pingshuo, Shanxi Province, China. The main results of this study are as follows: (1) In the horizontal direction, except for the strong variation (variation coefficient ≥ 100%) in soil organic matter (SOM) content at some depths, the degree of variation in other soil physicochemical properties at various depths was moderate or weak (variation coefficient < 100%). The soil physicochemical properties gradually remained constant after years of reclamation. In the vertical direction, the soil bulk density (SBD), soil porosity, SOM content, soil C/N ratio, soil total nitrogen (STN) content, soil available phosphorus (SAP) content, and soil available potassium (SAK) content showed significant variations (*p* < 0.05) between soil depths. In contrast, no significant difference was found for other physicochemical properties. (2) The SBD, STN, SAK, soil porosity, and soil clay content were significantly different (*p* < 0.05) under different vegetation cover types, but the influence of vegetation on other soil physicochemical properties seemed to be limited. The results reveal that trees have a stronger ability to reduce soil grain sizes and enhance SAP contents than shrubs or herbs; however, the beneficial effects of herbs on the physicochemical properties of shallow soil are more obvious than those of trees and shrubs. (3) This study indicates that more shrubs and trees should be planted in the areas with low vegetation coverage, and more measures should be taken to improve soil physicochemical properties in order to prevent the occurrence of large-scale degradation. The reconstruction of soil structure should be preferentially considered in the process of soil reconstruction and revegetation in areas under similar conditions. Herbs should first be planted in the early reclamation stage. At the same time, shrubs or trees can be adopted in the middle and late stages of vegetation reconstruction in order to achieve comprehensive revegetation.

## 1. Introduction

According to the “Statistical Review of World Energy 2020” [1], the world’s coal production in 2019 was 167.58 million tons, of which China’s coal production accounted for 47.63%, representing an increase of 0.94% from 2018. Opencast mining is a widely used method in the coal mining industries of many countries in the world, characterized by large mining capacity, fast construction speed, high labor efficiency, low production costs, and high resource recovery rates [2]. The coal mining area is the integrated region of coal mining, utilization, and land resource occupation and destruction [3], where land reclamation can exert a positive influence on improving local environmental conditions, which has become an increasingly hot topic all over the world. In August 1977, the United States Congress promulgated the first national land reclamation law, “Open-Pit Mining Management and Reclamation Law”, which unified and standardized the land reclamation process in American mining areas [4]. The “Prussian Mining Law” of Germany, issued in 1950, clearly requires the reclamation of the landscape for mining areas [5]. In 1989, China implemented the “Land Reclamation Regulations” [3], which prompted China to make great progress in land reclamation and ecological restoration. The present situation of opencast coal mining in China is characterized by small quantity and large capacity, super-large scale, and high concentration [6]. However, China’s coal reserves are mainly distributed in the northwest and north of China, most of which belong to the Loess Plateau region, where soil erosion is serious and the ecological environment is extremely fragile [7,8]. Opencast coal mining can trigger many adverse effects on the ecological environment, which can seriously damage the surface soil and vegetation [7]. Opencast coal mining can even disrupt the structure of underground aquifers and affect the normal recharge circulation path of groundwater [9], which can further cause large losses in vegetation and agricultural production [10]. Therefore, it is of great significance to maintain China’s fragile ecosystem via the artificial restoration of damaged land in mining areas.

Soil construction and revegetation are essential steps for ecological restoration in mining areas [11,12,13]. On the one hand, soil provides the necessary nutrients for plants, and directly affects their growth [14,15,16,17]; on the other hand, the physical structure and chemical properties of soil are also regulated by the processes of vegetation root growth (e.g., the absorption and release of nutrients) [18]. As essential indicators of soil quality, the properties of constructed soils become more heterogeneous due to the random dumping of soil during overburden placement and previous soil reclamation [13,19], making the physicochemical properties of reconstructed soils different from those of natural soils. Many scholars have studied the spatial distribution characteristics of soil physicochemical properties, and have revealed the spatial variation rules of soil physicochemical properties on reclaimed land [20,21,22,23,24,25]. The values of physicochemical properties of reconstructed soil are higher than those of natural soil [26], and different soil physicochemical properties are significantly correlated [12]. The distribution patterns of reconstructed soil pore are heterogeneous [7,27], and SBD shows a tendency to increase with soil depth [28,29,30]. The volumetric water content of reconstructed soil presents obvious spatial variations at different locations and at different depths of the same profile [31]. The pH value of topsoil is lower than that of the deeper soil [30], and soil organic carbon gradually decreases with the soil depth [32]. The mean values of SOM, STN, and soil C/N at 0–10 cm are generally higher than those at 10–20 cm [29]. Correlations between soil chemical properties tend to become weak with soil depth at 0–40 cm [33].

Additionally, the effects of different vegetation on soil properties are different, for which numerous studies have been carried out to explore the mechanisms by which vegetation cover types exert influence on the reconstructed soil properties in mining areas [14,15,34,35,36]. For example, the Chinese scholar Yao [37] found that forests have the strongest ability to improve the reconstructed soil quality, while the improvement of grass is very limited. Based on soil quality evaluation, Wang et al. [38] found that the growth of *Robinia pseudoacacia* can effectively improve soil quality. Hu et al. [39] found that revegetation had no significant effect on soil P, and that soil K could be preferentially improved by shrubs and herbs instead of trees. Mukhopadhyay et al. [40] applied the reclaimed mine soil index (RMSI) to analyze the physicochemical and biological properties of the rhizosphere soil of six common tree species growing in backfilled coal gangue; moreover, they analyzed the differences in soil nutrient concentrations under different tree species and compared the effects of reclamation by using these soils in reclaimed land [41]. Some scholars found that different kinds of vegetation have different effects on the soil nitrogen conversion rate [42]. The SOM under different vegetation configuration patterns is higher than that of the original soil [43,44]. The combination of different vegetation can control the surface runoff [45], and revegetation exerts a certain influence on the transformation processes of soil heavy metals, which can significantly improve soil quality [46,47].

In order to figure out the evolution laws of soil physicochemical properties in mining areas, it is essential to explore the spatial distribution differences in soil physicochemical properties, as well as the effects of vegetation cover on soil properties. However, most studies on reconstructed soil properties in mining areas are limited to surface soil, without exploring the influence of vegetation on soil properties at different depths [29,48]. In this study, we examine the differences in the vertical and horizontal distributions of reconstructed soil physicochemical properties in the south dump of the Antaibao opencast mine in Pingshuo Mine. Additionally, the differences in soil physicochemical properties under different vegetation cover types are also explored, providing theoretical support to the related research on the mechanisms of interaction between soil properties and vegetation. Overall, this study can provide some insight for the development of land reclamation strategies in mining areas.

## 2. Materials and Methods

### 2.1. Study Area

**Figure 1:**
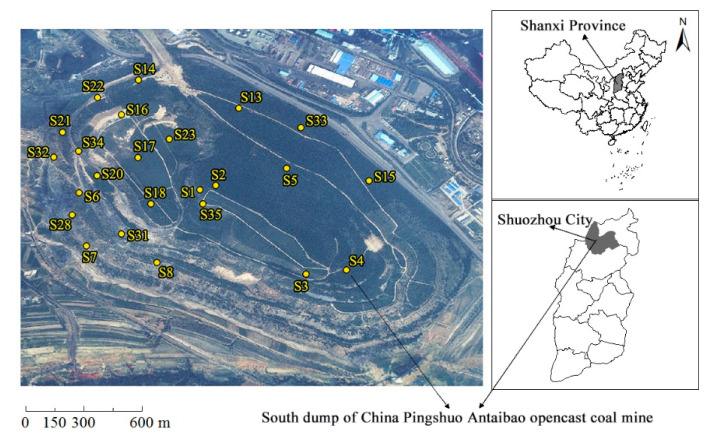
Locations of the study area and sample sites.

As of the end of the 20th century, Pingshuo Mine was the largest opencast coal production base in China, located in the east of the Loess Plateau and the north of Shanxi Province [49]. Located at the junction of Shuozhou City and Pinglu District in Shanxi Province (39°23′–39°37′ N, 112°10′–113°30′ E), Pingshuo Antaibao opencast coal mine covers a total area of 376 km^2^; it has coal reserves of ~12.6 billion tons, and the main coal type is gas coal. Antaibao opencast mine is located in the gentle slope of the Loess Plateau, which is characterized by an arid climate, sandy soils, poor water retention ability, and serious soil erosion. The study area (south dump of Antaibao opencast coal mine) is an early external dump; the dumping time was 1985–1989, the altitude is 1360–1465 m, and the slope is 20–40° [31]. The surface is covered with 100 cm of loess, laterite, and red loess. The reclamation of the study area began in 1989 [50], and the vegetation configuration mode of “trees, shrubs, and herbs” was adopted in order to artificially plant vegetation [51]. At present, the south dump has formed a multi-level and multi-type vegetation structure of trees, shrubs, and herbs; *Robinia pseudoacacia*, *Ulmus pumila*, *Pinus tabuliformis* and *Hippophae rhamnoides* are the main vegetation [38]. The ecological environment has been effectively restored. The locations of the study area and sample sites are shown in Figure 1.

### 2.2. Sample Data

A total of 24 sample sites in the study area were chosen for sample collection, and soil samples were separately collected in mid-May 2018 and mid-August 2018 (Figure 1). For the first sampling, the size of the large samples on the platform was 10 m × 10 m, which was adjusted according to the actual situation on the slope in order to ensure that the vertical projection of the sample was 10 m × 10 m [52]. For the second sampling, the size of the large samples on the platform was 5 m × 5 m, which was adjusted according to the actual situation on the slope in order to ensure that the vertical projection of the sample was 5 m × 5 m. Four corners of the large samples were fixed with wooden piles. Then, the longitude, latitude, and elevation of the samples’ center points were measured with GPS. The vegetation data were obtained through investigation of vegetation growth. Finally, a 1 m × 1 m sample was randomly selected from the large sample for the collection of herbs and the production of the soil profile [52].

### 2.3. Sampling and Testing

(1) Tree/shrub coverage measurement: Two diagonals of the large sample were selected for visual estimation of tree/shrub coverage, and then the average value was employed as the tree/shrub coverage;

(2) Herb sample collection: A small sample of 1 m × 1 m was randomly selected from each large sample square to collect herb samples on the surface. A total of 24 herb samples were collected, which were stored in sealed and numbered bags. Then, the herbs’ biomass was weighed after drying in a laboratory oven at 65 °C for ~10 h to constant weight;

(3) Soil sample collection: Soil profiles were dug at each sample site, and then soil samples were collected at the 10 cm interval with a ring knife (φ100). The average depth of soil profiles at each site was 80 cm; however, due to the difficulty of sampling and the shallow soil barrier layer at some sample sites, all sample sites (except for S7 and S15) were sampled to a depth of 60 cm below the surface [53]. A total of 138 soil samples were collected and put into freshness protection packages and sealed bags. A certain amount of soil at each layer was collected in cloth bags (a total of 138 soil samples) and sent to the Beijing Academy of Agriculture and Forestry Sciences for determination of other soil properties. In the laboratory, the collected ring soil samples were first weighed, and then baked in an oven at 105.50 °C for ~8 h before being weighed again at constant weight, and the bulk density and porosity of the soil were calculated. Except for soil bulk density and soil porosity, other soil properties were tested by the Beijing Academy of Agriculture and Forestry Sciences. 

### 2.4. Excluding Outliers

In order to ensure data accuracy, the Grubbs test was applied to exclude sample points containing outliers, for which 8 sample points were excluded and only 16 sample points (90 soil samples) were adopted in the following analyses (The original data volume was 24). Soil physicochemical properties in the study area are shown in Table 1.

### 2.5. Research Content

First, we analyzed the horizontal and vertical differences in reconstructed soil physicochemical properties in the study area by using descriptive statistical analysis and one-way ANOVA [54]. Then, we grouped samples by the growth conditions of vegetation in the study area. Single-factor variance analysis was used to research the differences in soil physicochemical properties under different vegetation cover types. Finally, we obtained the response relationship between vegetation and reconstructed soil physicochemical properties, and put forward some advice on soil reconstruction and revegetation practice.

## 3. Results

### 3.1. Horizontal Differences in Soil Physicochemical Properties

Microsoft Office Excel was used to calculate the variation coefficients of soil physicochemical properties at different depths. Then, we analyzed the horizontal differences in soil physicochemical properties. The results are shown in Table 2.

As shown in Table 2, the variation coefficients of SOM were the largest compared with that of the other reconstructed soil properties, and its variation range was also the most drastic. The SOM at 0–10 cm and 20–40 cm showed strong variation (variation coefficient ≥ 100%), while the SOM at 10–20 cm and 40–50 cm showed moderate variation (100% > variation coefficient ≥ 10%). Except for SOM, the variation coefficients of other soil physicochemical properties at all depths were less than 100%, showing medium or weak variation. The variation coefficients of soil pH were less than 10%, indicating weak variation. With the change in depth, the degree of variation of soil physicochemical properties also changes. The variation coefficients of soil pH and SAK decreased with the increase in soil depth. The variation coefficients of some soil physicochemical properties at 20–50 cm were significantly higher or lower than those at other depths. The variation coefficients of soil grain size content were characterized by similar variation tendency with the increase in soil depth. The variation coefficients of SBD and soil porosity increased with the increase in depth. The variation coefficients of SAP showed an increasing trend with the increase in depth, but were significantly lower at 40–50 cm than at other depths. The variation coefficients of ATN, SOM, and soil C/N showed a decreasing trend with the increase in depth, but the variation coefficient at 20–40 cm was significantly higher than those at other depths.

### 3.2. Vertical Differences of Soil Physicochemical Properties

Considering the limitation of the small sample size, the S–W test (Shapiro–Wilk test) was used to check the normality of soil physicochemical properties in IBM SPSS Statistics 22. The results showed that the original values of the soil physical properties, the reciprocals of STN, SOM, soil C/N, and the square root of soil pH, SAK, SAP conformed to normal distribution (*p* > 0.05). Therefore, the original values of the soil physical properties and the normalized values of the soil chemical properties were adopted for the following analyses. 

**Figure 2:**
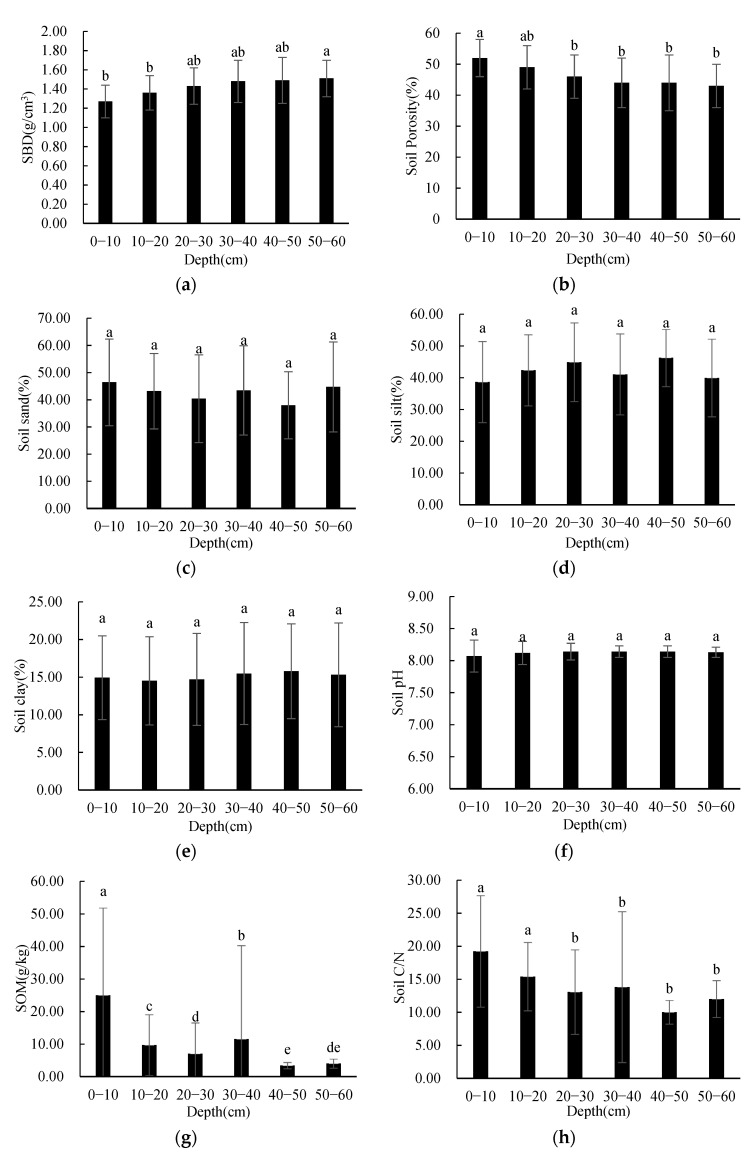

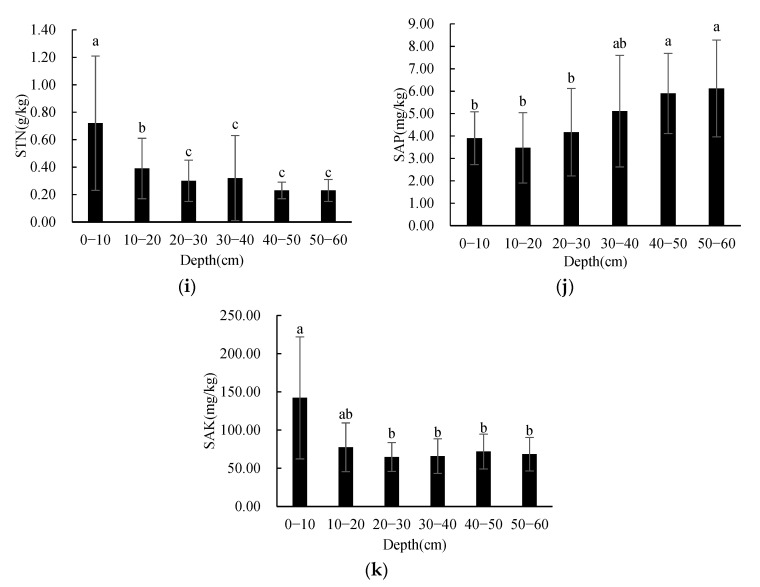
One-way ANOVA results of soil physicochemical properties at different depths (**a**–**k**) describe distribution characteristics of SBD (**a**), Soil Porosity (**b**), Soil Sand (**c**), Soil Silt (**d**), Soil Clay (**e**), Soil pH (**f**), SOM (**g**), Soil C/N (**h**), STN (**i**), SAP (**j**), SAK (**k**), respectively; different letters denote significant differences; *p* < 0.05; a > b > c > d).

In order to research the vertical differences in soil physicochemical properties in the study area, one-way ANOVA was employed in order to evaluate the differences in the soil physicochemical properties, with depth as the impact factor when the variance was homogeneous. Welch analysis was used when the variance was not homogeneous. The results showed that the SBD, soil porosity, SOM, soil C/N, STN, SAP, and SAK had significant differences between depths (*p* < 0.05). Other soil physicochemical properties had no significant difference between depths. The results are shown in Figure 2.

As shown in Figure 2, SBD and soil porosity had significant differences between 0–10 cm and 20–60 cm, and between 10–20 cm and 50–60 cm. The SBD tended to increase with soil depth, while soil porosity presented the opposite vertical distribution patterns. There was no significant difference in the three soil grain size contents between depths, and there was no obvious rule of change in the three soil grain size contents with the increase in soil depth. There was no significant difference in soil pH between depths, and the variation trend in soil pH was not obvious with depth. The SOM at 0–10 cm and 10–20 cm was significantly different from those at other depths, and the SOM at 20–30 cm was significantly different from those at 0–20 cm and 40–50 cm. The SOM tended to decrease with soil depth, but was significantly higher at 30–40 cm than those at other depths. Soil C/N showed significant differences between 0–10 cm and 20–60 cm, between 0–10 cm and 30–60 cm, and between 20–30 cm and 40–50 cm; soil C/N tended to decrease with soil depth. STN at 0–10 cm was significantly different from that at other depths, and at 10–20 cm was significantly different from that at 0–10 cm and 30–60 cm; STN tended to decrease with soil depth. SAP showed significant differences between 0–10 cm and 40–60 cm, between 10–20 cm and 30–60 cm, and between 20–30 cm and 40–60 cm; the SAP tended to increase with soil depth. SAK showed significant differences between 0–10 cm and 20–60 cm, and it tended to decrease with soil depth.

### 3.3. Differences in Soil Physicochemical Properties under Different Vegetation Cover Types

The 16 sample points were divided into three groups according to the growth conditions of vegetation in the study area (Table 3). There were 11 sample points with trees growing, sorted by the tree canopy closure, and divided into two groups on average; among them, those with tree canopy closure greater than 65% were classed as the “Trees—high canopy closure” group, set as Group 1; the sample points with tree canopy closure less than 65% were classed as the “Trees—low canopy closure” group, set as Group 2. All of the sample points with only shrubs and herbs growing were classed as the “Shrubs” group, and set as Group 3.

To research the differences in soil physicochemical properties under different vegetation cover types in the study area, we used one-way ANOVA to analyze soil physicochemical properties, with groups as the impact factor at each depth. The results showed that SBD and soil porosity had significant differences between groups at 30–60 cm; soil clay content had significant differences between groups at 30–50 cm; STN had significant differences between groups at 0–10 cm and 40–50 cm; and SAK had significant differences between groups at 0–50 cm. There was no significant difference in other soil physicochemical properties between groups at different depths.

**Figure 3:**
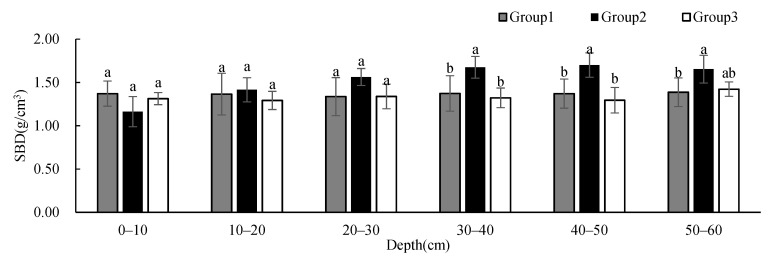
One-way ANOVA results of SBD under different vegetation cover types at different depths (different letters denote significant differences; *p* < 0.05; a > b).

**Figure 4:**
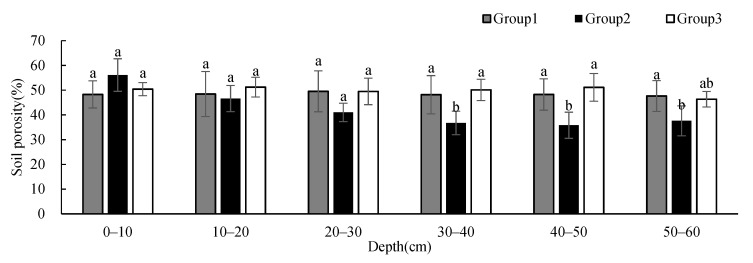
One-way ANOVA results of soil porosity under different vegetation cover types at different depths (*p* < 0.05; a > b).

The SBD (Figure 3) and soil porosity (Figure 4) in Group 2 were significantly different from those in Group 1 and 3 at 30–50 cm while those in Group 1 were significantly different from those in Group 2 at 50–60 cm. The BSD of all groups showed an increasing tendency with depth. The SBD of Group 2 presented much more obvious variations at 30–60 cm, and it was higher than that of the other two groups when the depth was below 20 cm. At the same time, soil porosity showed the opposite distribution patterns. 

**Figure 5:**
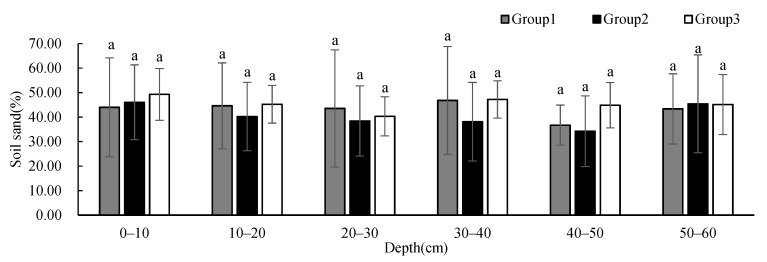
One-way ANOVA results of soil sand contents under different vegetation cover types at different depths (*p* < 0.05).

**Figure 6:**
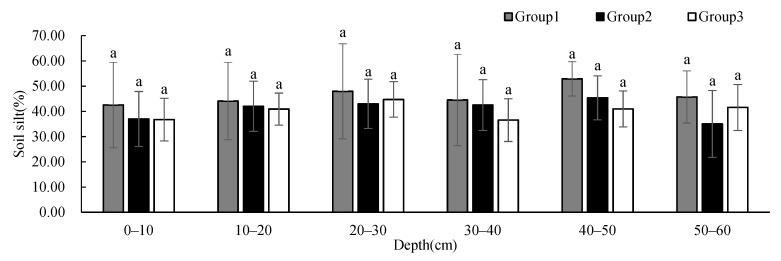
One-way ANOVA results of soil silt contents under different vegetation cover types at different depths (*p* < 0.05).

**Figure 7:**
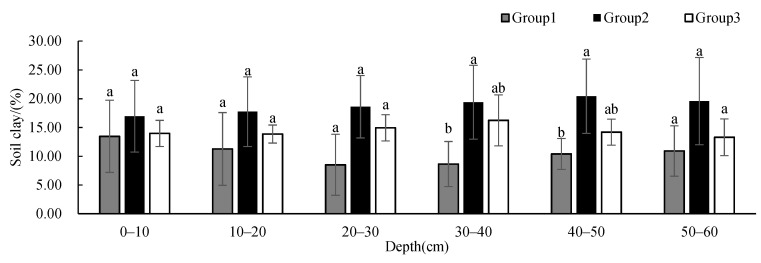
One-way ANOVA results of soil clay contents under different vegetation cover types at different depths (*p* < 0.05; a > b).

Soil sand (Figure 5) and silt (Figure 6) contents showed no significant difference between groups at different depths, and these contents remained relatively constant with the increase in depth. The soil sand content of Group 2 showed a decreasing trend with depth, and the content was less than that of the other two groups below 10 cm, but the content was significantly higher at 50–60 cm than those at other depths. The soil silt content of Group 1 was higher than that of the other two groups. The soil clay content (Figure 7) showed significant differences between Group 1 and Group 2 at 30–50 cm. The soil clay content of Group 1 first decreased and then increased with depth. The soil clay content of Group 2 showed an increasing trend with depth, while the soil clay content of Group 3 first increased and then decreased with depth. The soil clay content of Group 1 was significantly lower than that of the other two groups, and the soil clay content of Group 2 was significantly higher than that of the other two groups.

**Figure 8:**
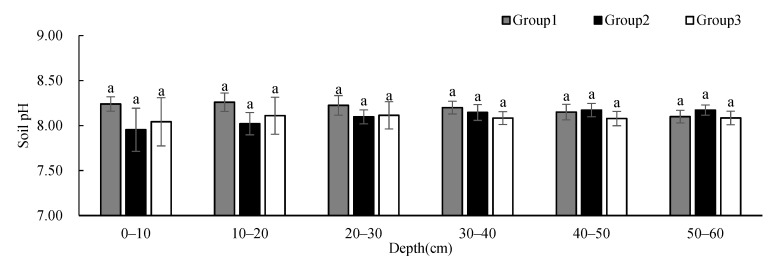
One-way ANOVA results of soil pH under different vegetation cover types at different depths (*p* < 0.05).

**Figure 9:**
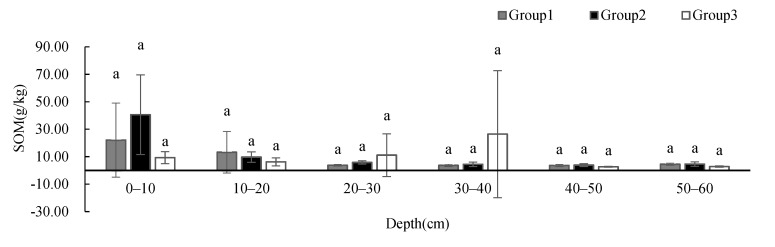
One-way ANOVA results of SOM under different vegetation cover types at different depths (*p* < 0.05).

**Figure 10:**
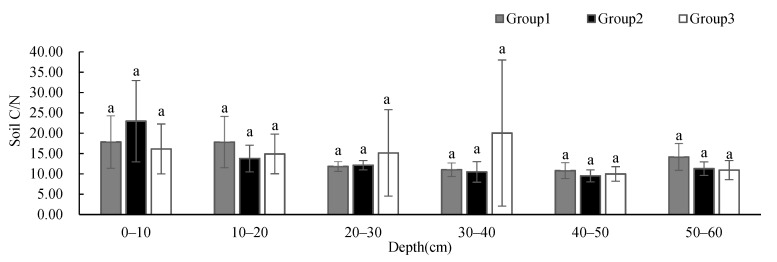
One-way ANOVA results of soil C/N under different vegetation cover types at different depths (*p* < 0.05).

Soil pH (Figure 8) showed no significant difference between groups at different depths, and the change in pH values is not obvious with the increase in depth. While the soil pH of Group 1 showed a slightly decreasing trend with depth, the soil pH of Group 2 showed an increasing trend with depth. Despite the large standard deviations, the SOM (Figure 9) showed no significant difference between groups at different depths, and showed a decreasing trend with depth. The SOM of Group 1 was higher than that of the other two groups at 0–10 cm, and was similar to that of the other two groups below 10 cm. The SOM of Group 3 was significantly higher at 20–40 cm than that of the other two groups. Soil C/N (Figure 10) showed no significant difference between groups at different depths, and its changing trend was the same as that of SOM. 

**Figure 11:**
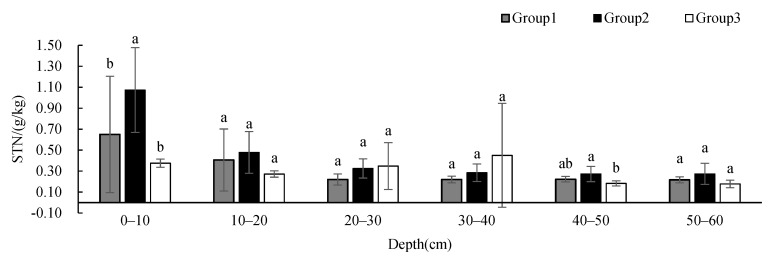
One-way ANOVA results of STN under different vegetation cover types at different depths (*p* < 0.05; a > b).

**Figure 12:**
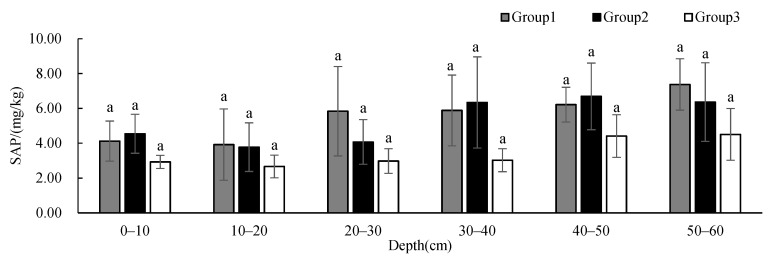
One-way ANOVA results of SAP under different vegetation cover types at different depths (*p* < 0.05).

**Figure 13:**
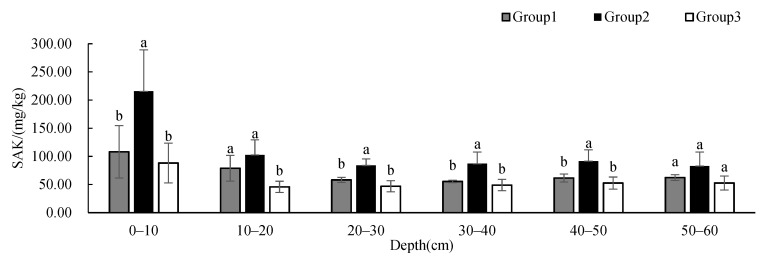
One-way ANOVA results of SAK under different vegetation cover types at different depths (*p* < 0.05; a > b).

The STN in Group 2 was significantly different from those in Group 1 and 3 at 0–10 cm while the STN in Group 2 was significantly different from that in Group 3 at 40–50 cm (Figure 11). The STN of all groups showed a decreasing tendency with depth, but the STN of Group 3 was significantly higher at 20–40 cm than that of the other two groups. The SAP (Figure 12) showed no significant difference between groups at different depths, and the content showed an increasing tendency with depth. The SAK in Group 2 was significantly different from those in Group 1 and 3 at 0–10 cm and 20–50 cm while the SAK in Group 2 was significantly different from that in Group 3 at 10–20 cm (Figure 13). The SAP of all groups showed a decreasing tendency with depth. The SAP of Group 1 and Group 2 was higher than that of Group 3 at all depths. The STN and SAP of Group 2 were significantly different from those of the other two groups.

## 4. Discussion

### 4.1. Analysis of Changes in Reconstructed Soil Physicochemical Properties

The random dumping of soil during mining activities in the mining area increases the heterogeneity of reconstructed soil properties, while land reclamation can reduce the spatial correlation of soil physicochemical properties and homogenize soil properties [13,55]. It has been more than 30 years since the reclamation started in the study area in 1989, and the soil physicochemical properties have remained constant after years of reclamation at various depths. Therefore, except for SOM, the variation coefficients of other soil physicochemical properties at the same depth in the study area all showed medium or weak variation. SOM is mainly derived from plant roots and surface vegetation (e.g., dead branches and leaves). The complicated distribution of surface vegetation causes the SOM in different regions of the study area to vary greatly. In addition, the variation coefficient of SOM is significantly higher at 20–40 cm than those at other depths in the study area, perhaps due to the presence of coal gangue in some sample sites. Some studies found that coal gangue possesses abundant nutrients, which can significantly improve the fertility of reclaimed soils [56]. The beneficial effect of coal gangue on SOM is more obvious than those on other soil physicochemical properties [57], causing the SOM at 20–40 cm to be significantly higher than those at other soil depths at some sample sites. The results of soil physicochemical properties in this study are consistent with those of Tan et al. [58], Zhao et al. [59], Yu et al. [28], Li et al. [60], and Xing et al. [61]. 

As for the soil physical properties in the study area, the soil particles were rearranged and the soil compactness increased due to the rolling of large machinery during reclamation [52,62,63], leading to the increase in SBD and the decrease in soil porosity [14,15,48]. Some studies found that the SBD tends to decrease while soil porosity tends to increase during land reclamation processes [7,64,65,66], which can also be confirmed by comparing the average soil physicochemical properties at 0–20 cm in the study area with those reported by other studies (Table 4). This study found that the SBD tended to increase with the increase in depth, while soil porosity tended to decrease with the increase in depth, which may also result from the land reclamation continuously reducing the soil bulk density. However, with the increase in soil depth, the soil improvement effect decreases significantly [58]. At the same time, some studies found that vegetation can improve soil structure and soil quality through the growth of roots, external organic matter input from litter, and root exudates [58,67]. Vegetation roots are mainly distributed in shallow soil [68], so the SBD and soil porosity of shallow soil are of higher quality relative to deep soil. The study area is located in the Loess Plateau, where soils are mainly composed of the particles of loess formed by weathering, and the soil texture is generally loam [60]. Therefore, this study found no obvious rule of change in the three soil grain size contents with the increase in soil depth. 

As for chemical properties in the study area, the soil pH was improved by the release of carbon dioxide through vegetation roots’ respiration, the secretion of protons and organic acids during the active absorption of ions by roots, and the elongation of root tip cells [71]. There was no significant difference in soil pH values between depths in the study area, and the variation tendency of soil pH with the increase in soil depth was not obvious compared with other soil physicochemical properties, possibly as a result of the land reclamation practice having been ongoing for more 30 years. Soil texture was relatively uniform at different depths, leading to little difference in soil pH. The difference in SOM at different depths in the study area was obvious, because the SOM is mainly provided by surface vegetation and other organisms, and the closer to the surface [39,58], the more organic matter is accumulated. Therefore, the content of SOM in shallow soil was higher than that in deep soil. Some studies have found that soil nitrogen mainly comes from the organic matter formed by the decomposition and synthesis of plant residues, and its dynamic changes are consistent with those of soil organic carbon [34,72,73,74]. Therefore, STN showed a decreasing tendency with the increase in depth. The value of soil C/N is inversely proportional to the decomposition rate of organic matter. A higher soil C/N value denotes a lower decomposition degree of organic matter, which is conducive to the accumulation of soil organic carbon [72,75]. In this study, the soil C/N decreased with the increase in depth, which may have been a result of the absorption rate of nitrogen by vegetation roots in shallow soil being faster than that in deep soil, resulting in a higher C/N in shallow soil relative to deep soil. As a kind of sedimentary element, soil phosphorus content decreases continuously due to adsorption by plants. In addition, the soil of the Loess Plateau is deficient in phosphorus, so the SAP in this study area increased with depth [38,61]. Microorganisms form a series of chelates and acid phenolic complexes when they decompose dead branches, promoting the decomposition of potassium minerals in the soil and increasing the content of soil available potassium [38]. The contents of microorganisms and dead branches in the shallow soil were higher than those in the deep soil, so SAK decreased with depth. As shown in Table 4, SAP in the study area was significantly lower than that in undamaged soil. Other soil physicochemical properties were improved compared with the soil after 3-year reclamation, and closer to those of undamaged soil, which can also support the results of our study.

In addition, some studies have found that due to the limitation of water in the early stage of revegetation in the Loess Plateau, herbs exert the highest effect on soil [58,65]. Group 2 had low tree canopy closure and higher herb biomass than that of the other two groups (Table 3); many physicochemical properties of Group 2 were significantly different from the other two groups. This may be due to the herbs’ planting density being greater than that of trees and shrubs. The beneficial effect of herbs on shallow soil in a small area is more obvious than those of trees and shrubs. In Group 2, the soil sand contents below 10 cm were less than those in the other two groups, while soil clay contents were greater than those in the other two groups. The results show that the effects of herbs on the reduction in SBD and the increase in soil porosity in the shallow soil are better than those of shrubs and trees, but in the deep soil, they are worse than those of shrubs and trees. The effect of trees on soil grain size reduction is worse than those of shrubs and herbs. The SOM of Group 1 was higher than that of the other two groups at 0–10 cm, and reduced to a similar content as that of the other two groups below 10 cm. This shows that the growth of trees can effectively improve the SOM in the surface soil, but not significantly improve the SOM in the deep soil. The SAP of Group 1 and Group 2 was higher than that of Group 3 at all depths; the STN and SAK of Group 2 were significantly different from those of the other two groups. This shows that the effect of herbs on the content of nitrogen and potassium in soil is more than that of trees and shrubs, and the beneficial effect of trees on SAP is better than that of shrubs and herbs. 

### 4.2. Guiding Significance for Reclamation Management of Mining Areas

According to the “Quality Control Standard of Land Reclamation in the Loess Plateau” (TD/T 1036-2013) and the “Nutrient Classification Standard of the Second National Soil Survey” [76], the quality of soil physicochemical properties in the study area was evaluated. It was found that the SBD of Group 2 did not reach the quality control standard of forest land reclamation in the Loess Plateau at 20–60 cm. The results show that the beneficial effect of herbs on the SBD of deep soil was worse than that of trees and shrubs. The SOM contents of the three groups did not reach the reclamation quality standard in the soil horizon below 40 cm, and the SOM contents of Group 1 at 20–40 cm and Group 2 at 30–40 cm also did not reach the standard. This shows that the beneficial effect of shrubs on SOM in deep soil is better than of by trees. Moreover, the SOM in the deep soil can still be greatly improved. Soil pH values in the study area all belonged to level 5. Except for the SOM and SAK of Group 2 at 0–10 cm, which belonged to level 1, the contents of SOM, STN, SAK, and SAP of all groups belonged to level 3 or below. The overall nutrient status of the soil in the study area is poor, which may also be caused by the poor nutrient status of the original soil [77]. Therefore, reclamation advice and countermeasures should not be formulated based on nutrient status alone, but should also consider the local situation. In the future, it will be necessary to pay attention to the regular monitoring of the areas with poor soil nutrient status, and to carry out technical research on the improvement of soil chemical properties in the study area, in order to ensure the sustainable development of the ecological environment.

In conclusion, for the study area, we suggest that the revegetation should be carried out according to local conditions, and that soil quality improvement should be carried out according to the soil physicochemical properties in different areas in the future. In areas where only trees grow (Group 1 and 2), shrubs could be appropriately replanted to improve the SOM of shallow soil. The area of Group 2 should be replanted with trees, such as elms, in order to improve SBD and increase regional vegetation coverage. Herbs should be replanted in the areas of Groups 1 and 3 in order to improve the fertility of shallow soil. In addition, coal gangue or other organic fertilizers can be used to improve the fertility of reclaimed soil. Finally, monitoring of each soil index of the dump should be strengthened in order to prevent large-scale degradation.

In the future, in areas with similar conditions, soil reconstruction and revegetation should first pay attention to the reconstruction of soil architecture. During soil reconstruction processes, it is necessary to ensure the uniformity of the soil in each area during dumping, and to reduce the differences in soil physicochemical properties. Given the differences in vertical distribution patterns, the shallow topsoil in the original land can be dumped separately in advance, and then be covered on the surface after the deep soil is dumped, in order to reduce the difficulty of revegetation and soil improvement in the later stages and improve the reclamation efficiency. At the same time, it is necessary to prevent the soil barrier layer from being set too shallow to affect the vegetation growth. The restoration of soil function should be emphasized during soil reconstruction processes, and it is a necessary role of microtopography construction in the restoration of soil function. Herbs should be mainly planted in the early stage of reclamation, and shrubs or trees suitable for the local environment should be introduced in the middle or late stages of revegetation, in order to realize the comprehensive revegetation modes of shrub grass, arboreal grass, or arboreal shrub grass, and improve the restoration of soil quality.

## 5. Conclusions

(1) In the horizontal direction, except for the strong variation in SOM at 0–10 cm and 20–40 cm, the degree of variation of other soil physicochemical properties at various depths was moderate or weak. The soil physicochemical properties gradually remained constant after years of reclamation. In the vertical direction, the SBD, soil porosity, SOM, soil C/N, STN, SAP, and SAK showed significant differences between depths; other physicochemical properties showed no significant difference between depths;

(2) Under different vegetation cover types, soil clay contents show significant differences between groups at 30–50 cm; the SBD and soil porosity show significant differences between groups at 30–60 cm; the STN shows significant differences between groups at 0–10 cm and 40–50 cm; and she SAK shows significant differences between groups at 0–50 cm. Other soil physicochemical properties had no significant difference between groups at different depths. The results show that trees have a stronger ability to reduce soil grain sizes and enhance SAP contents than shrubs or herbs. The beneficial effects of herbs on the physicochemical properties of shallow soil are more obvious than those of trees and shrubs;

(3) In the later management and conservation of the study area, more shrubs or trees should be planted in the areas with low vegetation coverage, and soil indices in some areas should be improved. Meanwhile, more environmental monitoring should be carried out regularly in order to prevent the large-scale degradation of the land. In the process of soil reconstruction and revegetation in areas with similar conditions, attention should be preferentially paid to the reconstruction of soil structure. At the same time, herbs should be mainly planted in the early stage of reclamation, and shrubs or trees should be adopted in the middle and late stages of vegetation reconstruction, in order to achieve comprehensive revegetation.

## Figures and Tables

**Table 1:** Soil physicochemical properties in the study area (*n* = 90).

Index	SBD g/cm^3^	Soil Porosity %	Soil Sand %	Soil Silt %	Soil Clay %	pH	SOM g/kg	C/N	STN g/kg	SAP mg/kg	SAK mg/kg
Average	1.42	46.42	42.77	42.13	15.10	8.12	10.41	14.06	0.37	4.73	82.63
Standard deviation	0.21	8.09	15.56	12.12	6.26	0.15	18.73	7.49	0.32	2.14	48.98
Range	1.10	41.48	68.11	57.66	29.62	0.96	116.81	49.17	1.62	8.62	285.71
Maximum	2.00	65.95	81.42	68.3	31.95	8.40	119.00	55.92	1.75	10.27	313.96
Minimum	0.90	24.46	13.31	10.64	2.33	7.44	2.19	6.76	0.13	1.65	28.25
Median	1.44	45.56	40.36	43.53	14.44	8.10	4.50	11.94	0.26	4.18	67.25
Variation coefficient (%)	15.10	17.43	36.37	28.77	41.46	1.88	179.94	53.31	85.54	45.24	59.28

SBD: soil bulk density; SOM: soil organic matter content; STN: soil total nitrogen content; SAP: soil available phosphorus content; SAK: soil available potassium content.

**Table 2:** Variation coefficients of soil physicochemical properties at different depths.

Depth cm	SBD %	Soil Porosity %	Soil Sand %	Soil Silt %	Soil Clay %	pH %	SOM %	C/N %	STN %	SAP %	SAK %
0–10	13.02	12.07	34.35	33.05	37.23	3.04	107.24	43.90	68.24	30.26	56.19
10–20	13.06	13.80	32.19	26.43	40.36	2.20	96.53	33.67	57.06	45.29	41.15
20–30	13.20	15.42	39.95	27.58	41.59	1.56	135.60	48.97	50.24	46.77	29.11
30–40	14.90	18.77	37.83	31.08	43.77	1.12	249.65	82.68	94.89	48.74	34.30
40–50	15.98	20.55	32.57	19.50	39.83	1.09	28.12	18.02	27.50	30.39	31.72
50–60	12.59	16.71	37.01	30.66	44.98	0.95	35.38	23.19	35.29	35.29	32.23

SBD: soil bulk density; SOM: soil organic matter content; STN: soil total nitrogen content; SAP: soil available phosphorus content; SAK: soil available potassium content.

**Table 3:** Grouping of sample points in the study area.

**Group**	**Description**	**Specific Sample**	**Dominant Vegetation Species**	**Vegetation Coverage (%)**	**Quantity of Trees/Shrubs**	**Herb Biomass (g)**
1	Trees—high canopy closure	S6, S8, S15, S20, S32	*Robinia pseudoacacia*, *Ulmus pumila*, *Pinus tabuliformis*	78.35	14.00	63.02
2	Trees—low canopy closure	S1, S4, S5, S14, S18, S33	*Robinia pseudoacacia*, *Ulmus pumila*	62.50	5.63	199.38
3	Shrubs	S3, S7, S17, S34, S35	*Caragana Korshinskii*	80.00	18.10	116.87

**Table 4:** Average soil physicochemical properties at 0–20 cm in the study area, and those reported by other studies.

Index	SBD g/cm^3^	Soil Porosity %	Soil Sand %	Soil Silt %	Soil Clay %	pH	SOM g/kg	C/N	STN g/kg	SAP mg/kg	SAK mg/kg
Undamaged [48]	1.30	50.94	-	-	-	8.11	9.68	13.10	0.50	6.32	90.60
3-Years-Reclamation [29,69,70]	1.55	41.51	28.33	58.41	13.27	8.13	4.17	10.85	0.26	6.01	123.10
Study area	1.32	50.26	44.79	40.49	14.72	8.10	17.36	17.31	0.56	3.69	109.79

SBD: soil bulk density; SOM: soil organic matter content; STN: soil total nitrogen content; SAP: soil available phosphorus content; SAK: soil available potassium content.

## References

[1] BP p.l.c (2020). BP Statistical Review of World Energy 2020.

[2] Tian H., Cai Q., Zhen X. (2014). Development Prospects of Surface Coal Mining Industry in China. Coal Eng..

[3] Bian Z. (2000). Research on land reclamation in coal mine area at China and abroad. China Land Sci..

[4] Schladweiler B.K. (2018). 40 years of the Surface Mining Control and Reclamation Act (SMCRA): What have we learned in the State of Wyoming. Int. J. Coal Sci. Technol..

[5] Jin D., Bian Z. (2009). Polices, Laws and Regulations on Land Reclamation and the Implications: Comparing China with Other Countries. China Land Sci..

[6] Li H., She C., Zhou Y., Huang Y. (2019). Summary and prospect of open-pit coal mining technology in China. China Land Sci..

[7] Qin Q., Wang J., Bai Z., Guo L., Wang H. (2016). Three-dimensional reconstruction and quantitative characterization of reconstruction soil pore at opencast coal mine dump based on CT scanning. J. China Coal Soc..

[8] Zhang Y., Wang J., Zhu Y. (2018). Effects of land subsidence caused by coal mining on the spatial variation of soil total nitrogen and organic matter concentrations in loess area. Chin. J. Ecol..

[9] Liu Q., Zhang Z., Zhang B., Mu W., Zhang H., Li Y., Xu N. (2021). Hydrochemical analysis and identification of open-pit mine water sources: A case study from the Dagushan iron mine in Northeast China. Sci. Rep..

[10] Pepliński B., Czubak W. (2021). The influence of opencast lignite mining dehydration on plant production—a methodological study. Energies.

[11] Haigh M.J., Gentcheva-Kostadinova S. (2002). Ecological erosion control on coal-spoil banks: An evaluation. Ecol. Eng..

[12] Wang H., Wang J., Cao Y., Lu Y., Qin Q., Wang Y. (2016). Effect of soil and topography on vegetation restoration in an opencast coal mine dump in a loess area. Acta Ecol. Sin..

[13] Feng Y., Wang J., Bai Z., Reading L. (2019). Effects of surface coal mining and land reclamation on soil properties: A review. Earth-Sci. Rev..

[14] Munkholm L.J., Schjonning P., Kay B.D. (2002). Tensile strength of soil cores in relation to aggregate strength, soil fragmentation and pore characteristics. Soil Tillage Res..

[15] Nawaz M.F., Bourrie G., Trolard F. (2013). Soil compaction impact and modelling. A review. Agron. Sustain..

[16] Qin Q., Wang H., Li X. (2018). Effect of Cutting on Forest Soil Function. World For. Res..

[17] Wu Y., Li B. (2006). Soil Science.

[18] Liu Q. (2016). The Coupling Relationship of Vegetation Patterns and Soil Properties in Typical Reach of The Middle and Lower Reaches of The Yellow River. Master’s Thesis.

[19] Zhao Y., Liu H., Wang X., Zou Y., Tian S. (2018). Research on relationship between terrain factors and soil physical properties of reclamation dump based upon UAV image. China Coal.

[20] Zhao L. (2013). Study on Land Reclamation Mode in Shuozhou Mining Area. Master’s Thesis.

[21] Sonn Y.K., Hur S.O., Hyun B.G., Cho H.J., Shin K.S. (2014). Differences in Spatial Variation of Soil Chemistry Between Natural and Anthropogenic Soils. Korean J. Soil Sci. Fertil..

[22] Jing M. (2014). Evolution, Water and Soil Response and Optimization of Landform Construction in Giant Open-pit Coal Mine on Loess Area. Ph.D. Thesis.

[23] Cao Y., Wang J., Bai Z., Zhou W., Zhao Z., Ding X., Li Y. (2015). Differentiation and mechanisms on physical properties of reconstructed soils on open-cast mine dump of loess area. Environ. Earth Sci..

[24] Min X., Li X., Li Q. (2017). Influence of mechanical compaction on reclaimed soil particle size distribution multifractal characteristics. Trans. Chin. Soc. Agric. Eng..

[25] Wang S., Cao Y., Bai Z., Luo G., Kuang X., Yang G. (2020). Spatial Characteristics of Reconstructed Soil Texture in Dumping Site of Loess Open-pit Mining Area. J. Northwest. For. Univ..

[26] Liu X., Bai Z., Zhou W., Cao Y., Zhang G. (2017). Changes in soil properties in the soil profile after mining and reclamation in an opencast coal mine on the Loess Plateau, China. Ecol. Eng..

[27] Zhang J.R., Wang J.M., Qin Q., Bai Z. (2017). Three-dimensional Multi-fractal Characteristics of Reconstruction Soil Pore at Opencast Coal Mine Dump based on CT Scanning. Chin. J. Soil Sci..

[28] Yu D., Jia X., Huang L., Shao M., Wang J. (2019). Spatial Variation of Soil Bulk Density in Different Soil Layers in the Loess Area and Simulation. Acta Pedol. Sin..

[29] Zhang G., Bai Z., Zhang C., Zhang J. (2015). Study on the Changes of Physic-chemical Properties of the Topsoil in Typical Plots in Pingshuo Mining Area. Hubei Agric. Sci..

[30] Schroeder P.D., Daniels W.L., Alley M.M. (2010). Chemical and Physical Properties of Reconstructed Mineral Sand Mine Soils in Southeastern Virginia. Soil Sci..

[31] Luo G., Cap Y., Bai Z., Kuang X., Wang S., Song L. (2019). Representation and Inversion of Reconstructed Soil Volumetric Water Content in Loess Open Pit Mining Area. J. Ecol. Rural. Environ..

[32] Liu W., Wag J., Bai Z., Zhang G. (2014). Soil Organic Carbon Dynamics of Reclaimed Soils at an Opencast Coal Mine. Met. Mine.

[33] Huang Y., Kuang X., Cao Y., Bai Z. (2018). The soil chemical properties of reclaimed land in an arid grassland dump in an opencast mining area in China. RSC Adv..

[34] Jobbágy E.G., Jackson R.B. (2000). The vertical distribution of soil organic carbon and its relation to climate and vegetation. Ecol. Appl..

[35] Kumar S., Singh A.K., Ghosh P. (2018). Distribution of soil organic carbon and glomalin related soil protein in reclaimed coal mineland chrono sequence under tropical condition. Sci. Total Environ..

[36] Li Y., Cao Y., Wang S., Luo G., Wang J., Zhou W., Bai Z. (2020). Changes of Typical Physical Properties of Reclaimed Mine Soil in the Dump Site of Loess Open Mining Area. Ecol. Environ. Sci..

[37] Yao X., Niu Y., Dang Z., Qin M., Wang K., Zhou Z., Zhang Q., Li J. (2015). Effects of natural vegetation restoration on soil quality on the Loess Plateau. J. Earth Environ..

[38] Wang Y., Zhao Z., Yuan Y., Guo A., Cao X., Li X. (2017). Evaluation of soil quality under three robinia pseudoacacia reclamation modes in Antaibao Opencast Mine. China Coal.

[39] Hu F., Du H., Zeng F., Song T., Peng W., Zhang F. (2018). Dynamics of soil nutrient content and microbial diversity following vegetation restoration in a typical karst peak-cluster depression landscape. Acta Ecol. Sin..

[40] Mukhopadhyay S., Maiti S.K., Masto R.E. (2013). Use of Reclaimed Mine Soil Index (RMSI) for screening of tree species for reclamation of coal mine degraded land. Ecol. Eng..

[41] Mukhopadhyay S., Masto R.E., Yadav A., George J., Ram L.C., Shukla S.P. (2016). Soil quality index for evaluation of reclaimed coal mine spoil. Sci. Total Environ..

[42] Yang Y., Ouyang Y., Chen H., Xiao K., Li D. (2018). Effects of Vegetation Restoration on Soil Nitrogen Pathways in a Karst Region of Southwest China. Environ. Sci..

[43] Zhang Y. (2014). Population Dynamics of Robinia pseudoacacia in Different Plant Configuration in Antaibao Opencast Coal Mine Dump. Master’s Thesis.

[44] Gu Y. (2017). Spatial variability and response relationship of vegetation restoration and soil water fertilizer in an opencast coal−mine dump in a loess area. Master’s Thesis.

[45] Wu J. (2017). Effects of landscape design on the construction of ecological agriculture demonstration garden. Chin. J. Agric. Resour. Reg. Plan..

[46] Sun C., Cheng Y., Wang X., Jiao L. (2017). Effects of vegetation restoration on soil physicochemical properties and heavy metal pollution characteristics in dump of open pit iron mine. Mod. Min..

[47] Chen J., Yang N. (2018). Soil Quality Assessment Along Re-vegetation on Sloping-Land with Purple Soils in Hengyang of Hunan Province, South-central China. Acta Agrestia Sin..

[48] Cao Y., Bai Z., Zhang G., Zhou W., Wang J., Yu Q., Du Z. (2013). Soil Quality of Surface Reclaimed Farmland in Large Open-cast Mining Area of Shanxi Province. J. Agro-Environ. Sci..

[49] Bai Z., Yun W. (2008). A case study on pingsshuo mining area: Land rehabilitation and reutilization in mining districts. Resour. Ind..

[50] Zhang H. (2002). Experience of Improving Surrounding Ecological Environment in Antaibao Open-pitCoal Mine. Opencast Coal Min. Technol..

[51] Li S. (2012). Land-use Change and Analysis of Land Reclamation Technology of An Taibao Opencast Mine. Master’s Thesis.

[52] Wang S., Cao Y., Luo G., Kuang X., Song L., Bai Z. (2019). Effects of reconstructing soil in open cast mining areas of the Loess Plateau on soil physical properties and vegetative growth. J. Agric. Resour. Environ..

[53] Luo G., Cao Y., Bai Z., Huang Y., Wang S. (2019). Soil bulk density difference, ground penetrating radar feature identification, and simulation for a reclaimed soil profile in the dumping site of an open pit mine. J. Agric. Resour. Environ..

[54] Liu M., Han G., Li X. (2021). Comparative analysis of soil nutrients under different land-use types in the Mun River basin of Northeast Thailand. J. Soils Sediments.

[55] Xiao B., Wang Q., Li C., Cao Z. (2011). Effects of reclamation of rehabilitated cropland on soil properties and its spatial variation on the Loess Plateau of China. J. Northwest. A F Univ. (Nat. Sci. Ed.).

[56] Zheng Y., Zhang Z., Yao D., Chen X. (2013). Study on Influence of Gangue on Reclaimed Soil Properties. J. Anhui Univ. Sci. Technol. (Nat. Sci.).

[57] Zhang Y., Song Z., Kong T., Zhao D., Wang L., Wang Y. (2021). Amelioration Effect of Coal Gangue on Physical and Chemical Properties of Saline-alkaline Soil. Ecol. Environ. Sci..

[58] Tan X., Mu X., Gao P., Sun W., Zhao G., Gu C. (2019). Effects of vegetation restoration on changes to soil physical properties on the loess plateau. China Environ. Sci..

[59] Zhao S., Zhao Y., Wu J. (2010). Quantitative analysis of soil pores under natural vegetation successions on the Loess Plateau. Sci. Sin. (Terrae).

[60] Li X., Tian J., Zhang C. (1992). A study on effects of different types of forest on the loess plateau on physical properties of soil. Sci. Silvae Sin..

[61] Xing H., Zhang J., Bai Z., Duan Y., Shangguan T., Guo D. (2015). Correlation Analysis on Plant Recovery and Soil Factors in Antaibao Opencast Coal Mine Dumps. Environ. Sci. Manag..

[62] Bradshaw A. (1997). Restoration of mined lands—Using natural processes. Ecol. Eng..

[63] Chen X., Hu Z., Zhang X. (2005). Reclamation Soil Compaction Spatial Variation Law about Different Reclamation Technologies in Subsidence Area. J. Irrig. Drain..

[64] Pang B., Yu Y. (2017). Analysis on Soil Microbial Community in Different Land Use Types on Reclamation of Coal Waste Pile. Chin. J. Soil Sci..

[65] Chang Q., An S., Liu J., Wang B., Wei Y. (1999). Study on Benefits of Recovering Vegetation to Prevent Land Deterioration on Loess Plateau. J. Soil Water Conserv..

[66] Wang J., Guo L., Bai Z., Qin Q., Lu C. (2016). Effects of land reclamation time on soil pore number and porosity based on computed tomography (CT) images in opencast coal mine dump. Trans. Chin. Soc. Agric. Eng..

[67] Gasch C., Huzurbazar S., Stahl P. (2014). Measuring soil disturbance effects and assessing soil restoration success by examining distributions of soil properties. Appl. Soil Ecol..

[68] Li T.C., Shao M.A., Jia Y.H. (2016). Application of X-ray tomography to quantify macropore characteristics of loess soil under two perennial plants. Eur. J. Soil Sci..

[69] Guo L., Wang J., Bai Z., Yang R., Cao Y. (2015). Analysis of spatial variability of soil granules in early stage of reclamation at opencast coal mine dump in loess area. China Min. Mag..

[70] Fan W., Li H., Bai Z. (2006). The Research of the Soil Fertility Changes in Loess Area Large Opencast Coal Mine’s Different Reclamation Models and Reclamation Years—Taking Pingshuo ATB Opencast Coal Mine as Example. J. Shanxi Agric. Univ. (Nat. Sci. Ed.).

[71] Hou J., Ye G., Zhang L. (2006). The Research Progress of Rhizosphere Soil of Forest Trees. Prot. For. Sci. Technol..

[72] Chen X., Liu H., Jiang S., Wang G. (2020). Effects of Reclamation Mode on Soil Aggregate Composition and Organic Carbon and Nitrogen Distribution in Mining Area. J. Shanxi Agric. Sci..

[73] Chen X., Li Z., Liu M., Jiang C. (2013). Effects of Different Fertilizations on Organic Carbon and Nitrogen Contents in Water-Stable Aggregates and Microbial Biomass Content in Paddy Soil of Subtropical China. Sci. Agric. Sin..

[74] Zhou S., Xiang Y., Xiao Y., Huang C., Tang J., Luo C., Han B., Liang K. (2017). Response of culturable soil microorganisms to simulated nitrogen deposition in a natural evergreen broadleaf forest in the Rainy Area of Western China. Acta Ecol. Sin..

[75] Schipper L.A., Sparling G.P. (2011). Accumulation of soil organic C and change in C:N ratio after establishment of pastures on reverted scrubland in New Zealand. Biogeochemistry.

[76] National Soil Census Office (1992). Soil Survey Technology in China.

[77] Wang J., Zhang M., Bai Z., Yang R., Guo L. (2014). Multi-fractal characteristics of reconstructed soil particle in opencast coal mine dump in loess area. Trans. Chin. Soc. Agric. Eng..

